# Medium-Frequency Neuromuscular Electrical Stimulation in Critically Ill Patients Promoted Larger Functional Capacity Improvement During Recovery than Low-Frequency Neuromuscular Electrical Stimulation: Randomized Clinical Trial

**DOI:** 10.3390/jcm14155407

**Published:** 2025-07-31

**Authors:** Pablo Guerra-Vega, Rodrigo Guzmán, Claudio Betancourt, Mario Grage, Cristian Vera, Macarena Artigas-Arias, Rodrigo Muñoz-Cofré, Kaio F. Vitzel, Gabriel Nasri Marzuca-Nassr

**Affiliations:** 1 Hospital Base San José Osorno, Osorno 1765, Chile; klgo.pabloguerra@gmail.com (P.G.-V.); klgo.rodrigoguzman@gmail.com (R.G.); claudiobet@hotmail.com (C.B.); mariojgrageb@gmail.com (M.G.); cristian.vera@clinicale.cl (C.V.); 2 Departamento de Ciencias de la Rehabilitación, Facultad de Medicina, Universidad de La Frontera, Claro Solar 115, Temuco 1145, Chile; klga.macarena.artigas@gmail.com; 3 Centro de Investigación e Innovación del Cáncer, Fundación Arturo López Pérez OECI Cancer Center, Santiago 0805, Chile; 4 Unidad de Enfermedades Respiratorias, Departamento de Medicina Interna, Facultad de Medicina, Universidad de La Frontera, Claro Solar 115, Temuco 1145, Chile; rodrigomunozcofre@gmail.com; 5 School of Health Sciences, College of Health, Massey University, Auckland 0632, New Zealand; k.vitzel@massey.ac.nz

**Keywords:** critical illness, rehabilitation, physical therapy, physical function

## Abstract

**Background/Objectives:** This study aimed to compare the effects of low- and medium-frequency NMES, combined with a standard physical therapy (SPT) program, on functional capacity in critically ill patients. **Methods:** Fifty-four critically ill patients admitted into Intensive Care Unit (ICU) and on mechanical ventilation participated in this randomized, single-blinded, experimental study. Participants were randomly assigned to a Control group, who received a lower limb SPT program; the Low-frequency NMES group received lower limb SPT + NMES at 100 Hz; and the Medium-frequency NMES group received lower limb SPT + NMES at 100 Hz with a carrier frequency of 2500 Hz. The outcomes, encompassing functional capacity in the hospital, included muscle strength, handgrip strength, functional status, degree of independence for activities of daily living, functional and dynamic mobility, quality of life, and total days hospitalized. **Results:** Both NMES protocols combined with SPT improved functional capacity compared to the control group. Medium-frequency NMES provided additional benefits on dynamic balance, in the degree of independence to perform activities of daily living and quality of life (all *p* < 0.001) prior to hospital discharge. It also promoted larger gains on functional status prior to ICU discharge and on knee extension strength (both *p* < 0.05) prior to intermediate care unit discharge. Medium-frequency NMES also enhanced handgrip strength earlier than low-frequency NMES when compared to the control group. Notably, medium-frequency NMES was the only intervention associated with a significant reduction in total hospital stay duration (*p* < 0.05). **Conclusions:** Medium-frequency NMES, along with an SPT program in critically ill patients, showed greater benefits on functional capacity during recovery than low-frequency NMES. (Trial registration: This trial is registered on ClinicalTrials.gov: NCT05287919). **Implications for rehabilitation:** 1. Medium-frequency NMES may enhance physical functionality and quality of life in critically ill patients with ICU-acquired weakness. 2. Medium-frequency NMES can reduce the number of hospitalization days. 3. NMES combined with SPT represents a feasible and effective option for patients unable to engage in active rehabilitation during critical illness.

## 1. Introduction

Critical illnesses are characterized by symptoms such as fever, hyperadrenergic activity, and a hypermetabolic state driven by systemic inflammation [1,2]. These conditions lead to progressive impairment of physiological functions, with increased muscle proteolysis and, consequently, muscle wasting [3,4]. Evidence indicates that within 24–36 h of sepsis onset, muscle regeneration is already compromised [5]. This impairment is characterized by myofibril atrophy, calcification of necrotic myofibrils, endomysial inflammation and fibrosis, and a reduced satellite cell pool [6]. Furthermore, skeletal muscle mass loss represents a significant complication in patients admitted to intensive care units (ICU), with muscle weakness being its most prominent clinical manifestation [7]. This weakness, coupled with diminished functional capacity, is referred to as ICU-acquired weakness (ICU-AW) [8,9].

ICU-AW is a multifactorial condition affecting approximately 46% of critically ill patients, particularly those exposed to medications such as corticosteroids, sedatives, or neuromuscular blocking drugs, as well as those requiring prolonged mechanical ventilation (MV) for seven days or more and extended periods of immobilization [10]. This condition is characterized by substantial skeletal muscle mass loss, predominantly in the lower limbs, driven by factors including severe inflammation, sepsis, multiple organ failure, and prolonged sedation [11]. These alterations are not only responsible for the decrease in the functional capacity of the patient but also influence clinical outcomes, such as duration of hospitalization and quality of life [12,13]. Furthermore, ICU-AW imposes a considerable economic burden on healthcare systems due to the costs associated with prolonged treatment and rehabilitation [14]. The pathophysiology of ICU-AW mechanisms includes structural alterations in skeletal muscle, impaired vascular supply, and mitochondrial dysfunction, which compromise the regenerative capacity of muscle tissue [15]. Consequently, ICU survivors frequently experience persistent physical, psychological, and cognitive sequelae, placing them at higher risk of long-term disability and reduced survival rates compared to patients in other hospital settings [16].

Long-term, ~25% to 50% of patients requiring MV in the ICU will go on to develop “post-intensive care syndrome,” a collection of sequelae that greatly impact functional capacity and health-related quality of life [17]. Prolonged hospitalization further exacerbates this functional decline, often persisting for years after discharge [18]. Consequently, it is essential for ICU survivors to regain muscle strength, mobility, and pre-admission functionality [19]. This need has become even more evident in the post-COVID-19 era, as recent evidence indicates that functional limitations and disability may persist for extended periods after ICU discharge, reinforcing the importance of implementing early and effective rehabilitation strategies in the context of critical illness [20].

Among the rehabilitation strategies to combat skeletal muscle atrophy, early mobilization stands as a cornerstone intervention, demonstrating significant improvements in short-term functional outcomes and contributing to long-term recovery of ICU patients [21,22,23,24]. In recent years, the utilization of physiotherapy approaches to address skeletal muscle weakness and wasting has been increasingly emphasized [25,26,27,28].

Neuromuscular electrical stimulation (NMES), a technique that induces visible muscle contractions through portable devices connected to surface electrodes [27], has emerged as an effective modality for treating muscle deterioration [29]. In clinical settings, NMES is commonly classified based on the carrier frequency of the current. Low-frequency NMES refers to biphasic pulsed currents delivered directly at frequencies ranging from 1 to 1000 Hz (normally using between 1 to 200 Hz), without a carrier signal. In contrast, medium-frequency NMES uses a continuous sinusoidal current with a carrier frequency between 1000 and 10000 Hz, modulated in low-frequency bursts [30]. A widely used example is the Russian current, which employs a 2500 Hz carrier modulated at 100 Hz. This type of waveform is associated with lower skin impedance, promoting deeper current penetration and improved patient tolerance [31]. In this study, we define low-frequency NMES as biphasic pulsed current at 100 Hz, and medium-frequency NMES as burst-modulated current with a 2500 Hz carrier.

It has been reported that NMES has physiological effects for maintaining muscle mass in critically ill patients regardless of patient involvement [32]. Specifically, low-frequency NMES has shown promise in attenuating skeletal muscle mass loss in ICU patients [23,33,34]. Nevertheless, the overall impact of low-frequency NMES in enhancing functional capacity in ICU patients remains inconclusive, likely due to protocol variability and patient heterogeneity [35,36,37].

On the other hand, medium-frequency NMES has demonstrated superior outcomes in preserving skeletal muscle mass in atrophic models [38], such as patients with chronic heart failure [22] and chronic obstructive pulmonary disease [21]. This superiority is thought to stem from higher tetanic force production and peak torque associated with increased stimulation frequency, which amplifies the NMES-induced stimulus on skeletal muscle mass [28]. However, no studies have yet explored the effects of different NMES frequency protocols on the functional capacity of ICU patients. Based on this evidence, it is hypothesized that medium-frequency NMES could result in greater preservation of functional capacity compared to low-frequency NMES in critically ill patients.

In accordance with the above, the aim of this study was to compare the effects of low-frequency versus medium-frequency NMES, when combined with a standard physical therapy (SPT) program, on the functional capacity of critically ill patients.

## 2. Materials and Methods

### 2.1. Design

This randomized, single-blind experimental study was approved by the Scientific Ethics Committee of the Valdivia Health Service and authorised by the Assistance Ethics Committee of the Hospital Base San José de Osorno (ORD No. 523). The trial was registered on ClinicalTrials.gov with the identifier NCT05287919. Informed consent was obtained from legal representatives at ICU admission and confirmed in writing by patients once they regained decision-making capacity.

### 2.2. Participants

In total, 160 patients of the Hospital Base San José de Osorno, Chile, were recruited and 54 were selected for the study between August 2019 and January 2020 (Supplemental Figure S1), based on the following inclusion criteria: patients aged 18–80 years, admitted to the ICU and requiring MV for more than 72 h. The exclusion criteria were as follows: non-sedated patients; spinal cord injury, cerebrovascular accident, presence of pacemakers, history of deep vein thrombosis, pregnancy, cardiac complications (e.g., history of myocardial infarction or congenital diseases), use of neuromuscular blockers, and polytraumatized patients requiring external fixation devices.

The nature and risks of the interventions were detailed to family members of each patient upon ICU admission, prior to obtaining authorization for the procedures. The cohort was randomly divided into three groups by an independent collaborator using the randomization tool from the website randomizer.org: Control group (CONTROL; 53 ± 18 years, BMI 25.8 ± 2.6 kg·m^2^, APACHE II 31 ± 2, n = 17), received only the standard physical therapy (SPT) program (passive mobilization) twice a day; low-frequency NMES group (NMES LFG; 62 ± 11 years, BMI 27.9 ± 2.8 kg·m^2^, APACHE II 33 ± 3, n = 18), submitted to low-frequency NMES and SPT twice a day; medium-frequency NMES group (NMES MFG; 59 ± 16 years, BMI 27.1 ± 1.7 kg·m^2^, APACHE II 31 ± 3, n = 17), submitted to medium-frequency NMES and SPT twice a day. Interventions concluded when sedation cessation procedures were initiated.

Functional capacity was assessed through measurements of muscle strength (Medical Research Council Sum Score, MRC-SS), handgrip strength, and functional status (Functional Status Score for the Intensive Care Unit, FSS-ICU) as soon as the patient awoke from sedation. These parameters were reassessed upon discharge from the ICU, the Intermediate Care Unit (IMCU), and hospital discharge. Additionally, dynamic balance (Timed Up and Go Test, TUG) and independence in activities of daily living (Barthel Index) were evaluated at both IMCU and hospital discharge. The Short Form 36 (SF-36) quality of life questionnaire was also administered at the time of hospital discharge. The number of days on MV and total hospital stay duration were recorded (Supplemental Figure S2).

Medical diagnoses, Acute Physiology and Chronic Health Evaluation II (APACHE II) scores, and Sequential Organ Failure Assessment (SOFA) scores were obtained upon ICU admission. Additionally, body mass index (BMI), medical history (prior diseases and surgeries), pharmacological treatments, hemodynamic data during the experimental procedures, and fluid balance were documented. This study was conducted as a single-blind trial, in which the outcome evaluators were blinded to group allocation. All assessments were performed by independent evaluators who were not involved in delivering the intervention. Standardized protocols were followed to ensure consistency and minimize the risk of observer bias.

### 2.3. Intervention

All SPT and NMES procedures were performed by a physiotherapist with extensive clinical experience in critically ill patients and NMES application. This professional was responsible solely for executing the intervention according to the established protocol, ensuring consistency across all participants, without involvement in study design or data interpretation. All interventions (SPT and NMES, depending on group allocation) were initiated within the first 24 h of ICU admission, once clinical and hemodynamic stability was confirmed.

Information on ongoing pharmacological treatments, as well as the patient’s ventilatory and hemodynamic status, was obtained prior to each intervention and considered during passive mobilization and NMES sessions to monitor the patient’s response.

#### 2.3.1. Standard Physical Therapy

All participants have received SPT sessions based on a passive range of motion mobilization protocol for the lower limbs [27]. This protocol consisted of a bilateral series of 10 repetitions of hip flexion, knee flexion and extension, and ankle flexion and extension. The procedure was performed twice daily, with one session in the morning (between 8:00 AM and 12:00 PM) and another in the afternoon (between 2:00 PM and 6:00 PM).

#### 2.3.2. Low- and Medium-Frequency Electrical Stimulation

Electrical stimulation was performed twice daily following the SPT sessions, maintaining the same schedule of one session in the morning (between 8:00 AM and 12:00 PM) and one in the afternoon (between 2:00 PM and 6:00 PM). Two electrodes were attached to each thigh at the motor points of the quadriceps muscle, based on a previous study [26]. The midpoint between the anterior superior iliac spine and the base of the patella was used as a reference, with the electrodes placed 15 cm apart—5 cm proximal and 10 cm distal to the reference point. After the first measurement, semi-permanent markers were used to indicate electrode positions, ensuring accurate and consistent placement in every session.

Electrical stimulation was performed using a 4-channel device (Sonopuls 492, series 4, Enraf-Nonius^®^, Rotterdam, The Netherlands). The low-frequency protocol consisted of a rectangular biphasic current at 100 Hz with 400 µs width pulses, delivered in trains of 5 s ON (ramp-up time: 1 s, plateau: 3 s, ramp-down time: 1 s) and 10 s OFF. The medium-frequency protocol followed similar parameters (rectangular biphasic current with the same train profile) but used a carrier frequency of 2500 Hz and a burst frequency of 100 Hz.

Each session lasted 20 min, resulting in a total of 40 min per day. The electrical current amplitude (mA) was adjusted to produce visible and palpable muscle contractions and was recalibrated every 3 min to maintain the initial contraction level. Initial and final amplitudes were recorded.

To ensure patient safety, sessions were monitored for potential adverse events, and interruptions were documented, including reasons such as hemodynamic instability or medical contraindications.

## 3. Outcome Measures

### 3.1. Clinical Assessment of Muscle Strength

Muscle strength was evaluated using the Medical Research Council-Sum Score (MRC-SS) [12,39]. Measurements were conducted once the patient awoke from sedation in the ICU, achieved a score of ≥3/5 on the S5Q scale, and prior to discharge from the ICU, IMCU, and hospital.

### 3.2. Handgrip Strength

Handgrip strength was measured using a digital dynamometer (JAMAR, Patterson Medical, Nottinghamshire, UK). The isometric force was measured only when patients were able to follow at least three out of five straightforward instructions, demonstrating their ability to cooperate and level of consciousness prior to the test, and if they scored ≥3 at S5Q [40]. The hospital bed was adjusted to a 90° upright position so that patients were seated with their elbows flexed at 90°. They were instructed to firmly hold the device and squeeze it with maximum strength for 3 s. The procedure was repeated three times, with a 30 s interval between attempts. The highest value recorded among the three attempts was used for analysis [41]. Measurements were performed once the patient awoke from sedation in the ICU and prior to discharge from the ICU, IMCU, and hospital.

### 3.3. Functional Status

The physical functioning of the patients was evaluated using the Functional Status Score for the Intensive Care Unit (FSS-ICU). Assessments were conducted once the patient awoke from sedation in the ICU and prior to ICU discharge [42].

### 3.4. Timed Up and Go Test

Functional mobility and dynamic balance were assessed using the Timed Up and Go (TUG) Test. This test measures the required strength for a patient to rise from a seated position with their back supported by the chair’s backrest and hands resting on their thighs, walk 3 m, turn around, walk back to the chair, and return to the initial seated position [43]. Measurements were conducted prior to IMCU and hospital discharges.

### 3.5. Barthel Index

The Barthel Index was used to estimate the degree of independence in activities of daily living based on the level of assistance required by the patient to complete specific tasks [44]. Measurements were conducted prior to IMCU and hospital discharges.

### 3.6. Quality of Life

Patient’s overall quality of life was evaluated using the Short Form 36 (SF-36) [45], which includes questions addressing physical and emotional status, social functioning, and general health. Measurements were conducted prior to hospital discharge.

### 3.7. Patient Clinical Registry

The following information was recorded daily for each patient: pharmacological treatments, laboratory test results, fluid balance, and hemodynamic data before and after the experimental procedures. Additionally, the number of days spent on MV, in the ICU, and in the hospital was documented.

## 4. Statistical Analysis

### 4.1. Power Calculation and Sample Size

The sample size was estimated based on the total MRC score for the lower right limb of ICU patients, following the results reported by Karatzanos et al. (2012) [46]. The calculation was performed using G*Power (v.3.1.9.2) with repeated-measures ANOVA, considering between-group effects (usual care, medium-frequency NMES, and low-frequency NMES) and within-group effects (measurements at different hospitalization time points). Hedges’ g was used to minimize potential bias. The effect size was 0.669, with a pre-post correlation of 0.50. A minimum of 15 participants per group was required to achieve 80% power (β = 0.20) with a significance level of α = 0.05.

Considering a 15% dropout rate, the final sample size was adjusted to 18 participants per group, totaling 54 participants.

### 4.2. Data Analysis

Data was presented as mean ± SD and analysed using GraphPad Prism 8 (GraphPad Software, San Diego, CA, USA). For normally distributed data, one-way ANOVA followed by Bonferroni’s post hoc test was applied for comparisons at a single time point. For comparisons across multiple time points, two-way repeated measures ANOVA followed by Bonferroni’s post hoc test was used. In figures, error bars represent the standard error of the mean (SEM). Differences were considered statistically significant at *p* < 0.05. Outliers were identified using Grubbs’ test (graphpad.com/quickcalcs/Grubbs1.cfm accessed on 7 April 2020).

## 5. Results

### 5.1. Participants Characteristics

From a cohort of 160 recruited patients admitted to the ICU of Hospital Base San José de Osorno, 54 were included in the study (Supplemental Figure S1). Two patients died due to the severity of their conditions on admission diagnosis. One polytrauma patient developed cardiorespiratory arrest on day 12 in the ICU, and another patient suffering from abdominal sepsis died on day 11 in the ICU after a decision to limit therapeutic interventions. Their admission data, clinical records, and intervention data up to the time of death were included in the results. After random allocation of participants into the three experimental groups, no significant baseline differences were observed (Table 1). Throughout the intervention period, NMES current amplitude was individually adjusted to elicit visible and palpable quadriceps contractions. Across all patients who received NMES, the mean initial amplitude was 47.4 mA (range: 18.6–77.5 mA), and the mean final amplitude was 55.8 mA (range: 20.5–85.2 mA). These values reflect the expected variability in critically ill patients and remain within effective and safe stimulation ranges.

### 5.2. Admission Diagnosis, Comorbidities and Pharmacological Treatment

The most common admission diagnosis was septic shock (12 patients; 22.2%), followed by severe sepsis (11 patients; 20.3%), abdominal diseases (10 patients; 18.5%) and respiratory diseases (9 patients; 16.6%). Other causes of admission included psychiatric disorders (4 patients; 7.4%), polytrauma (3 patients; 5.5%); circulatory diseases (2 patients; 3.7%); renal failure (2 patients; 3.7%) and neurological surgery (1 patient; 1.8%). Hypertension was the most frequent comorbidity (64%), followed by Diabetes Mellitus (50%), dyslipidaemia (38%), cardiac diseases (24%), renal diseases (22.2%) and arthrosis (14.8%).

Regarding pharmacological treatment, all participants received sedation, vasoactive drugs and other routine medication (anticoagulants, analgesics, gastrointestinal protectants). Antibiotics were administered to 70% of the patients (11 from CONTROL, 14 from LFG and 13 from MFG), while 54% received corticoids (8 from CONTROL, 11 from LFG and 10 MFG) and 31.4% of the patients received bronchodilators (associated with the admission diagnosis of respiratory disease).

### 5.3. Muscle Strength and Handgrip Strength

The overall muscle strength of patients assessed using the MRC-SS score, knee extension and handgrip strength, gradually increased after each evaluation time point in comparison with the initial measurement at ICU awakening, regardless of experimental group (*p* < 0.001). The force improvement elicited by NMES was not different between NMES LFG and NMES MFG, but the MRC-SS score of the MFG was the only one significantly higher than CONTROL across all time points (*p* < 0.05 at awakening in ICU and prior to ICU discharge; *p* < 0.01 prior to IMCU discharge; *p* < 0.001 prior to hospital discharge). The MRC score for knee extension was higher in both NMES groups when compared with CONTROL at all time points (*p* < 0.001). The only significant difference between NMES LFG and NMES MFG was prior to IMCU discharge, when NMES MFG displayed higher MRC-knee extension values than NMES LFG (*p* < 0.05) (Table 2).

The prevalence of ICUAW was 100% in all groups at ICU awakening and remained unchanged in CONTROL until IMCU discharge, with a reduction to 53% prior to hospital discharge. In contrast, the NMES LFG showed a gradual decrease in ICUAW prevalence, dropping to 89% before IMCU discharge and 33% prior to hospital discharge. Notably, the NMES MFG demonstrated the most significant recovery, with ICUAW prevalence decreasing to 88% before IMCU discharge and further reduced to only 6% prior to hospital discharge. These results highlight the effectiveness of NMES, particularly medium-frequency NMES, in mitigating ICUAW and promoting functional recovery (Table 2).

Handgrip strength of NMES LFG was significantly higher than CONTROL only at the last measurement (prior to hospital discharge) (*p* < 0.05), while the NMES MFG values were higher than CONTROL since the second measurement (*p* < 0.05 prior to ICU discharge; *p* < 0.01 prior to IMCU discharge; *p* < 0.001 prior to hospital discharge). However, there was no difference between the NMES groups.

### 5.4. Functional Status

**Figure 1:**
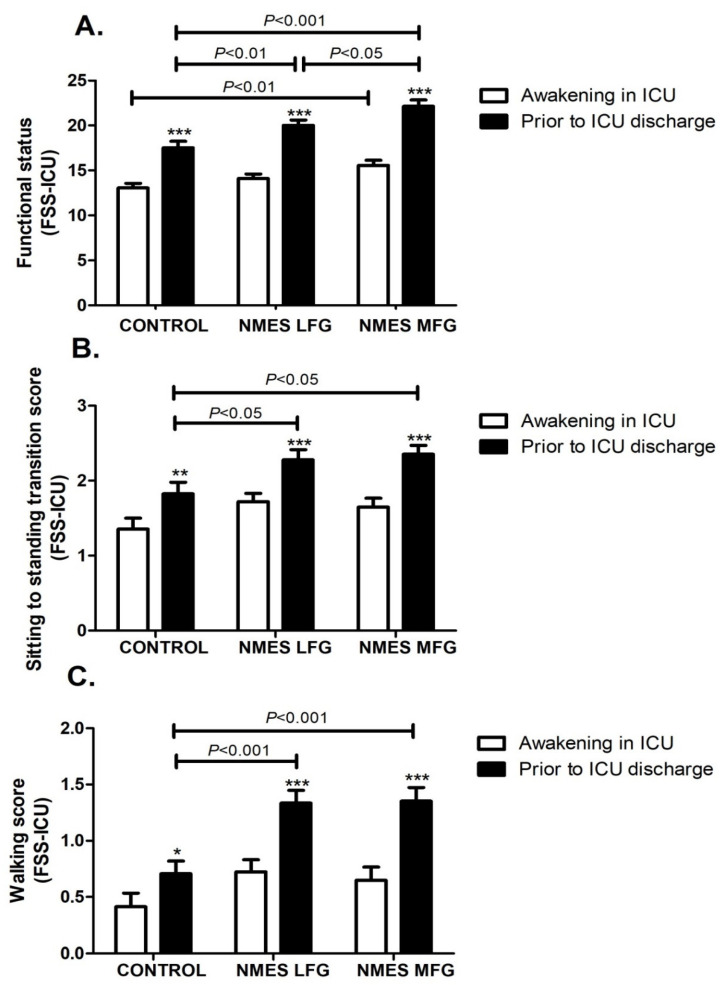
Functional Status Score for the Intensive Care Unit (FSS-ICU). (**A**) Overall score. (**B**) “Sitting to standing” subcategory score. (**C**) “Walking” subcategory score. *: *p* < 0.05, **: *p* < 0.01, and ***: *p* < 0.001 within the same group. n = 17 for Control group, n = 17–18 for LFG and n = 16–17 for MFG. ICU: Intensive Care Unit. LFG: low-frequency group. MFG: medium-frequency group. NMES: neuromuscular electrical stimulation.

Physical functioning, as measured by the FSS-ICU, improved significantly in the NMES MFG compared to the CONTROL as early as the moment patients awoke in the ICU (CONTROL: 13.1 ± 2.1, LFG: 14.1 ± 2.0, MFG: 15.6 ± 2.3; *p* < 0.01 for MFG vs. CONTROL). As patients were recovering in the ICU, all groups showed an increase in FSS-ICU values, measured again prior to ICU discharge (*p* < 0.001), in comparison to the initial assessment, with further beneficial effects from NMES. Both the LFG and MFG groups presented higher FSS-ICU scores prior to ICU discharge when compared with CONTROL. Moreover, MFG values were also significantly higher than LFG (CONTROL: 17.5 ± 3.0, LFG: 20.0 ± 2.5 and MFG: 22.1 ± 2.8; *p* < 0.01 for LFG vs. CONTROL; *p* < 0.001 for MFG vs. CONTROL; *p* < 0.05 for LFG vs. MFG) (Figure 1A). Breaking down the FSS into its subcategories, all three groups showed an improvement in the “sitting to standing” score while in ICU (awakening in ICU vs. prior to ICU discharge; CONTROL: *p* < 0.01, LFG and MFG: *p* < 0.001) and both the LFG and MFG groups were significantly higher than CONTROL in the final measurement, prior to ICU discharge (*p* < 0.05) (Figure 1B). The “walking” score has increased significantly by the end of ICU stay (awakening in ICU vs. prior to ICU discharge; *p* < 0.05 for CONTROL; *p* < 0.001 for LFG and MFG), with both NMES protocols providing further improvement and higher “walking” scores than CONTROL prior to ICU discharge (*p* < 0.001 for LFG or MFG vs. CONTROL) (Figure 1C).

### 5.5. Functional Mobility and Dynamic Balance

When this assessment first took place prior to IMCU discharge, both NMES groups displayed better (lower) results in the TUG test when compared with CONTROL (*p* < 0.01 for LFG vs. CONTROL; *p* < 0.001 for MFG vs. CONTROL), with MFG values being even lower than LFG (*p* < 0.01). For the final test (prior to hospital discharge), all groups have improved their times (*p* < 0.001), but the initial differences persisted: LFG times were significantly lower than CONTROL at the final TUG test (*p* < 0.01), while the MFG times were lower than both CONTROL (*p* < 0.001) and LFG (*p* < 0.001) (Table 3).

### 5.6. Independence for Activities of Daily Living

**Figure 2:**
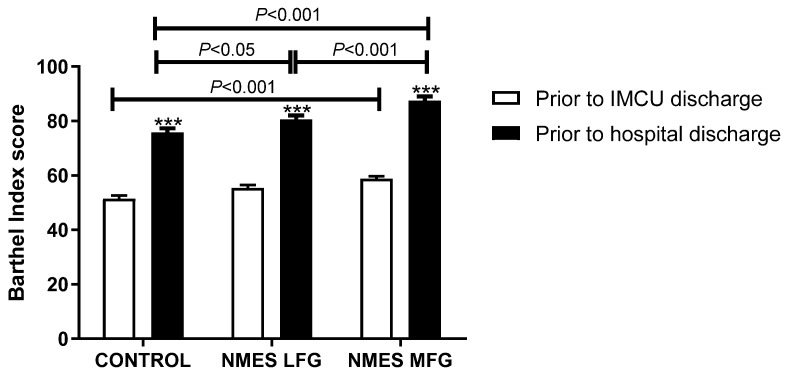
Barthel Index prior to IMCU and hospital discharges. ***: *p* < 0.001 within the same group. n = 17 for Control group, n = 17 for NMES LFG and n = 16 for NMES MFG. IMCU: Intermediate Care Unit. LFG: low-frequency group. MFG: medium-frequency group. NMES: neuromuscular electrical stimulation.

The Barthel Index was used as an estimate of the level of assistance required by the patients to perform common tasks. All groups have improved their score between IMCU and hospital discharges (CONTROL: 51.5 ± 4.6 to 75.9 ± 6.2, LFG: 55.4 ± 4.8 to 80.6 ± 6.1 and MFG: 58.8 ± 3.9 to 87.5 ± 6.1; *p* < 0.001). However, only the MFG scores were significantly higher than CONTROL already at the first measurement, at IMCU discharge (*p* < 0.001). Later, the measurements prior to hospital discharge showed additional improvements by both LFG and MFG, with higher scores than CONTROL (*p* < 0.05 and *p* < 0.001, respectively). Additionally, MFG values were also higher than LFG at hospital discharge (*p* < 0.001) (Figure 2).

### 5.7. Quality of Life

**Figure 3:**
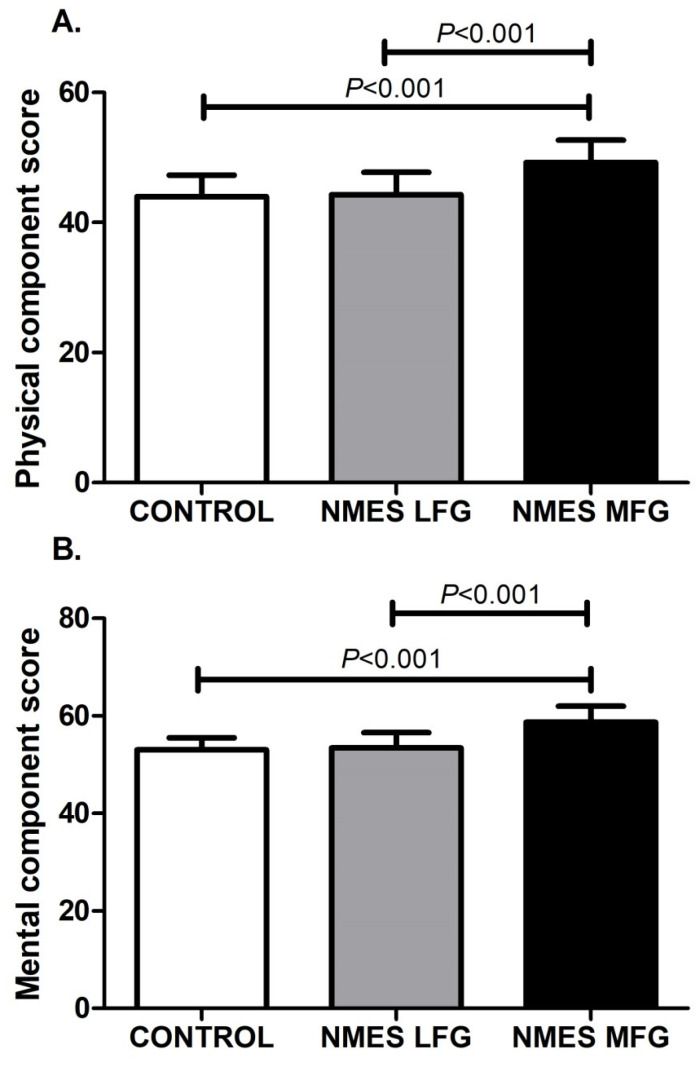
Physical and mental dimensions of overall quality of life evaluated by the Short Form 36 (SF-36) score prior to hospital discharge. (**A**) Physical component score. (**B**) Mental component score. n = 17 for Control group, n = 18 for NMES LFG and n = 17 for NMES MFG. LFG: low-frequency group. MFG: medium-frequency group. NMES: neuromuscular electrical stimulation.

Health-related quality of life was evaluated prior to hospital discharge based on the Physical Component Summary (PCS) and Mental Component Summary (MCS) of the SF-36 score. PCS values were CONTROL: 44.0 ± 3.3, LFG: 44.3 ± 3.4 and MFG: 49.2 ± 3.5. MCS values were CONTROL: 53.1 ± 2.5, LFG: 53.4 ± 3.1 and MFG: 58.7 ± 3.3. The LFG values were no different from CONTROL; however, the MFG scores were significantly higher than CONTROL, both for the PCS and MCS (*p* < 0.001) (Figure 3).

### 5.8. Physical Therapy Sessions and Days in MV, ICU and in Hospital

Both medium- and low-frequency NMES were generally well tolerated by participants, and no serious adverse events were reported.

There was no difference between groups in the number of days spent in MV (CONTROL: 8.8 ± 2.0, LFG: 8.6 ± 2.0 and MFG: 7.8 ± 2.5; *p* = 0.34) and in ICU (CONTROL: 11.5 ± 1.6, LFG: 11.1 ± 2.3 and MFG: 10.1 ± 3.0; *p* = 0.21). However, total hospitalization duration was CONTROL: 27.0 ± 4.7, LFG: 26.8 ± 4.8 and MFG: 25.8 ± 3.9 days, being significantly lower for MFG when compared with CONTROL (*p* < 0.05). CONTROL received 10.6 ± 3.2 sessions of SPT, while LFG and MFG received 9.4 ± 3.3 and 11.1 ± 4.3 sessions of SPT + NMES, respectively (*p* = 0.40).

## 6. Discussion

The study aimed to compare the effects of low- versus medium-frequency NMES, combined with a standard physical therapy (SPT) program, on the functional capacity of critically ill patients. We hypothesized that regular NMES sessions would improve functional capacity, with the medium-frequency protocol yielding the most significant effects.

Critically ill patients undergo a rapid process of skeletal muscle atrophy. Among other factors, it depends on admission diagnosis, pharmacological treatment received, and duration of ICU stay. There is an imbalance favoring muscle protein degradation over synthesis, associated with myofibrillar necrosis, macrophage infiltration, fibrosis and ectopic accumulation of fat [47]. Those alterations were described after 7–10 days of ICU admission, affecting up to 40% of the patients [47,48]. Skeletal muscle atrophy in critically ill patients leads to a significant decline in strength and functional capacity, resulting in greater dependence on performing basic daily activities, even after hospital discharge [13]. This persistent dysfunction compromises long-term recovery and the patients’ quality of life [49]. Therefore, it is essential to implement early interventions, such as early mobilization and tailored physiotherapy programs, to mitigate muscle atrophy and improve functional capacity post-discharge [24].

NMES is particularly relevant during the acute phases of illness, especially when critically ill patients under sedation exhibit no voluntary muscle contractions and are unable to actively engage in physical therapy exercises [50]. Previous studies have demonstrated that both low- and medium-frequency NMES, combined with a SPT program, are equally effective strategies for counteracting the ICUAW [51] and skeletal muscle atrophy of the lower limbs previously described in critically ill patients admitted to the ICU [38,48,52].

The effectiveness of NMES on functional capacity in critically ill patients remains a controversial topic, with inconsistent findings in the literature [39,53,54]. Nonoyama T. et al. (2022) conducted a retrospective study evaluating the early application of low-frequency NMES (20 Hz), initiated within 48 h of ICU admission, in combination with early mobilization in older adults [55]. The results of that study did not show significant differences in FSS-ICU scores, handgrip strength, or MRC sum scores at ICU or hospital discharge compared to a group that received standard therapy alone. However, these findings should be interpreted with caution, considering that older adults often exhibit anabolic resistance, a condition that limits adaptations in skeletal muscle mass and function [56,57].

In contrast, our study included a more diverse population, comprising both younger and older participants, and employed a medium-frequency NMES protocol (using a 2500 Hz carrier modulated at 100 Hz), which is recognized for its effectiveness in mitigating muscle atrophy, particularly in the quadriceps [58]. This methodological difference could account for the disparities observed in the results.

On the other hand, the early application of low-frequency NMES (80 Hz) combined with early mobilization has demonstrated benefits in reducing hospital length of stay. Campos R. et al. (2022) reported a significant reduction in the duration of hospitalization among critically ill patients who received SPT + NMES (18.5 days) compared to those who received SPT alone (30 days) [59]. Our results also showed a reduction in hospital days, although the lengths of stay observed in our study were longer than those reported by Campos R. et al. This discrepancy might be attributed to differences in the protocols, particularly the daily duration of the intervention, as Campos R. et al. (2022) [59] applied NMES for 60 min per day, whereas our protocol consisted of 40 min sessions.

Although frequencies above 200 Hz have been employed in NMES protocols with favorable results in mitigating skeletal muscle loss, not all studies have demonstrated consistent improvements in functional capacity [34]. For instance, Tsuchikawa Y. et al. (2024) evaluated the application of NMES to upper and lower limbs in patients with severe COVID-19 on MV and found no significant differences in MRC sum scores, handgrip strength, FSS-ICU scores, or ICU length of stay compared to the standard care group [60].

A possible explanation for these findings is the delayed initiation of NMES, which was applied on average 3.2 ± 1.4 days after ICU admission. At this point, muscle proteolysis was likely already established, limiting the intervention’s potential to prevent muscle loss or improve functionality [47]. This highlights the critical importance of early NMES initiation, ideally within the first 24–48 h of admission, to maximize its benefits.

The variability in outcomes reflects the inherent complexity of implementing NMES in critically ill patients. Factors such as the timing of initiation, stimulation frequency, session duration, and individual patient characteristics are key elements influencing the effectiveness of this intervention. Despite these inconsistencies, the demonstrated benefits in physical well-being [51,61], muscle strength, and functional status—evaluated using tools like the FSS-ICU and Barthel Index—underscore the potential of NMES [53,62,63]. The results obtained with both low- and medium-frequency protocols in this study highlight their value as a fundamental component of strategies aimed at optimizing functional recovery in this population.

In this context, our study demonstrated that the medium-frequency NMES protocol provided significant additional benefits compared to the low-frequency protocol. These improvements included better dynamic balance (assessed through the TUG test) and enhanced quality of life (SF-36) before hospital discharge, as well as increases in overall muscle strength (MRC-SS), physical functioning during intensive care (FSS-ICU), and independence in activities of daily living (Barthel Index) prior to discharge from the intermediate care unit and the hospital.

A notable finding was the earlier and more sustained improvement in handgrip strength with the medium-frequency protocol, observed before ICU discharge, whereas such progress was only achieved at hospital discharge with the low-frequency protocol. Handgrip strength, previously associated with lower mortality, shorter hospital stays, and better functional outcomes [64,65,66], may explain why the medium-frequency NMES protocol was the only intervention capable of significantly reducing the total hospital length of stay, from ICU admission to final discharge.

Though methodological limitations, such as variability in the cause of admission, comorbidities, and medications administered during hospitalization, could have influenced the results, random allocation and similar baseline APACHE II and SOFA scores between the control and experimental groups helped minimize this potential bias. Nonetheless, the findings should be interpreted with caution, as the NMES intensity was individually adjusted for each patient. Amplitude was defined as the minimum current required to produce a visible and palpable muscle contraction and was periodically modified during each session to adapt to the patient’s condition.

This study has several strengths, including its randomized design, standardized rehabilitation protocol, and the use of validated outcome measures such as the MRC-SS, FSS-ICU, Barthel Index, and SF-36. Moreover, the early initiation of NMES and the inclusion of both low- and medium-frequency protocols allowed for a more comprehensive evaluation of their differential effects on functional recovery in critically ill patients.

Although the study was single center with a modest sample size (n = 54), the number of participants was based on an a priori power analysis (effect size = 0.669, α = 0.05, power = 80%), ensuring sufficient statistical robustness. This design supports the internal validity of our findings, which provides a solid basis for future multicenter studies in broader ICU populations.

Moreover, although the overall rehabilitation protocol was standardized, patients progressed through the various stages of rehabilitation based on their individual advancements. This may have led to differences in the time and intensity dedicated to each phase, potentially impacting the results. The implementation of NMES in large muscle groups, combined with its early initiation—within the first 24 h of ICU admission—emerges as a key factor in optimizing the benefits of the intervention and improving functional outcomes.

While post-discharge follow-up (30–90 days) was not included in this study, due to institutional and logistical constraints during the COVID-19 period, our findings provide a relevant snapshot of in-hospital functional recovery. Future studies should aim to incorporate longer-term follow-up to assess the sustained impact of NMES beyond discharge.

In conclusion, the medium-frequency NMES protocol significantly improved functional capacity, muscle strength, and quality of life during hospitalization, surpassing the benefits observed with low-frequency NMES. These findings underscore the potential of NMES as an integral tool in the functional recovery of critically ill patients.

## Figures and Tables

**Table 1:** Baseline characteristics of participants.

	CONTROL (n = 17)	NMES LFG (n = 18)	NMES MFG (n = 17)	*p* Value
Women	9	6	8	
Men	8	12	9	
Age (years)	53 ± 18	62 ± 11	59 ± 16	0.205
Height (cm)	164 ± 6.1	165 ± 8.3	166 ± 6.6	0.654
Weight (kg)	70 ± 8.2	76 ± 10.4	75 ± 8.1	0.099
BMI	25.8 ± 2.6	27.9 ± 2.8	27.1 ± 1.7	0.054
APACHE II	31 ± 2	33 ± 3	31 ± 3	0.182
SOFA	14 ± 1	14 ± 1	15 ± 2	0.117

APACHE II: Acute Physiology and Chronic Health Evaluation II; BMI: body mass index; NMES: neuromuscular electrical stimulation; NMES LFG: low-frequency NMES group; NMES MFG: medium-frequency NMES group; SOFA: Sequential Organ Failure Assessment.

**Table 2:** Muscle strength and handgrip strength recovery during hospitalization ^1^.

	Awakening in ICU	Prior to ICU Discharge	Prior to IMCU Discharge	Prior to Hospital Discharge
**Control group**				
MRC-SS	33.3 ± 1.8	36.9 ± 1.7	41.4 ± 2.1	46.0 ± 2.5
ICUAW, n (%)	17 (100%)	17 (100%)	17 (100%)	9 (53%)
Knee extension (MRC)	5.5 + 0.7	6.5 ± 0.7	6.8 ±0.8	7.5 ± 0.7
Handgrip strength (kg)	9.0 ± 0.9	9.9 ± 0.9	11.6 ± 1.0	13.7 ± 0.9
**NMES LFG**				
MRC-SS	35.3 ± 2.7	38.7 ± 3.0	42.7 ± 3.2	48.2 ± 3.2
ICUAW, n (%)	18 (100%)	18 (100%)	16 (89%)	6 (33%)
Knee extension (MRC)	6.4 ± 0.5 ***	7.4 ± 0.5 ***	7.8 ± 0.7 ***	8.4 ± 0.5 ***
Handgrip strength (kg)	9.3 ± 1.2	10.7 ± 1.5	12.6 ± 1.6	14.9 ± 1.9 *
**NMES MFG**				
MRC-SS	35.6 ± 2.9 *	39.6 ± 2.8 *	44.5 ± 2.9 **	49.9 ± 3.0 ***
ICUAW, n (%)	17 (100%)	17 (100%)	15 (88%)	1 (6%)
Knee extension (MRC)	6.6 ± 0.5 ***	7.6 ± 0.5 ***	8.3 ± 0.7 ***^,#^	8.9 ± 0.5 ***
Handgrip strength (kg)	9.8 ± 1.2	11.2 ± 1.2 *	13.3 ± 1.5 **	15.9 ± 2.1 ***

Values presented as mean ± SD. ^1^: all parameters have increased significantly in comparison with the initial measurement (awakening in ICU) and any other previous measurement within the same group (*p* < 0.001). *: *p* < 0.05, **: *p* < 0.01 and ***: *p* < 0.001 vs. Control group. ^#^: *p* < 0.05 vs. NMES LFG. n = 17 for Control group, n = 17–18 for NMES LFG and n = 16–17 for NMES MFG. ICU: Intensive Care Unit. IMCU: Intermediate Care Unit. LFG: low-frequency group. MFG: medium-frequency group. MRC-SS: Medical Research Council-Sum Score. ICUAW: ICU acquired muscle weakness (MRC < 48). NMES: neuromuscular electrical stimulation.

**Table 3:** Recorded times of TUG tests performed prior to IMCU and hospital discharges ^1^.

	Prior to IMCU Discharge	Prior Hospital Discharge
**Control group**		
TUG test (s)	26.5 ± 2.4	22.5 ± 2.5
**NMES LFG**		
TUG test (s)	23.8 ± 2.5 *	20.0 ± 2.4 *
**NMES MFG**		
TUG test (s)	21.1 ± 1.8 **^,#^	16.8 ± 1.8 **^,##^

Values presented as mean ± SD. ^1^: all parameters have improved (decreased) significantly in comparison with the initial measurement (prior to IMCU discharge) within the same group (*p* < 0.001).*: *p* < 0.01 and **: *p* < 0.001 vs. Control group. ^#^: *p* < 0.01 and ^##^: *p* < 0.001 vs. NMES LFG. n = 17 for Control group, n = 18 for NMES LFG and n = 16 for NMES MFG. IMCU: Intermediate Care Unit. LFG: low-frequency group. MFG: medium-frequency group. NMES: neuromuscular electrical stimulation. TUG: Timed Up and Go.

## Data Availability

The datasets used and/or analyzed during the current study are available from the corresponding author on reasonable request.

## Supplementary Materials

The following supporting information can be downloaded at: https://www.mdpi.com/article/10.3390/jcm14155407/s1, Supplemental Figure S1. CONSORT Flowchart displaying participant recruitment, randomization and data analysis. BMI: body mass index; NMES: neuromuscular electrical stimulation; ICU: Intensive Care Unit. Supplemental Figure S2. Experimental design: ICU intervention and follow-up stages. FSS-ICU: Functional Status Score for the Intensive Care Unit; ICU: Intensive Care Unit; IMCU: Intermediate Care Unit; SPT: standard physical therapy; CONTROL: only SPT (group); LFG: low-frequency (group); MFG: medium-frequency (group); MRC-SS: Medical Research Council-Sum Score; MV: mechanical ventilation; NMES: neuromuscular electrical stimulation; S5Q: Standardized Five Questions; SF-36: Short Form 36; TUG: Timed Up and Go Test.
