# Supplementary material for: Medium-Frequency Neuromuscular Electrical Stimulation in Critically Ill Patients Promoted Larger Functional Capacity Improvement During Recovery than Low-Frequency Neuromuscular Electrical Stimulation: Randomized Clinical Trial
Source: J Clin Med. 2025 Jul 31;14(15):5407. doi: 10.3390/jcm14155407 (PMC12347956; doi:10.3390/jcm14155407)
Supplement: Supplementary file 1 [file jcm-14-05407-s001.zip › jcm-3779818-supplementary.pdf]

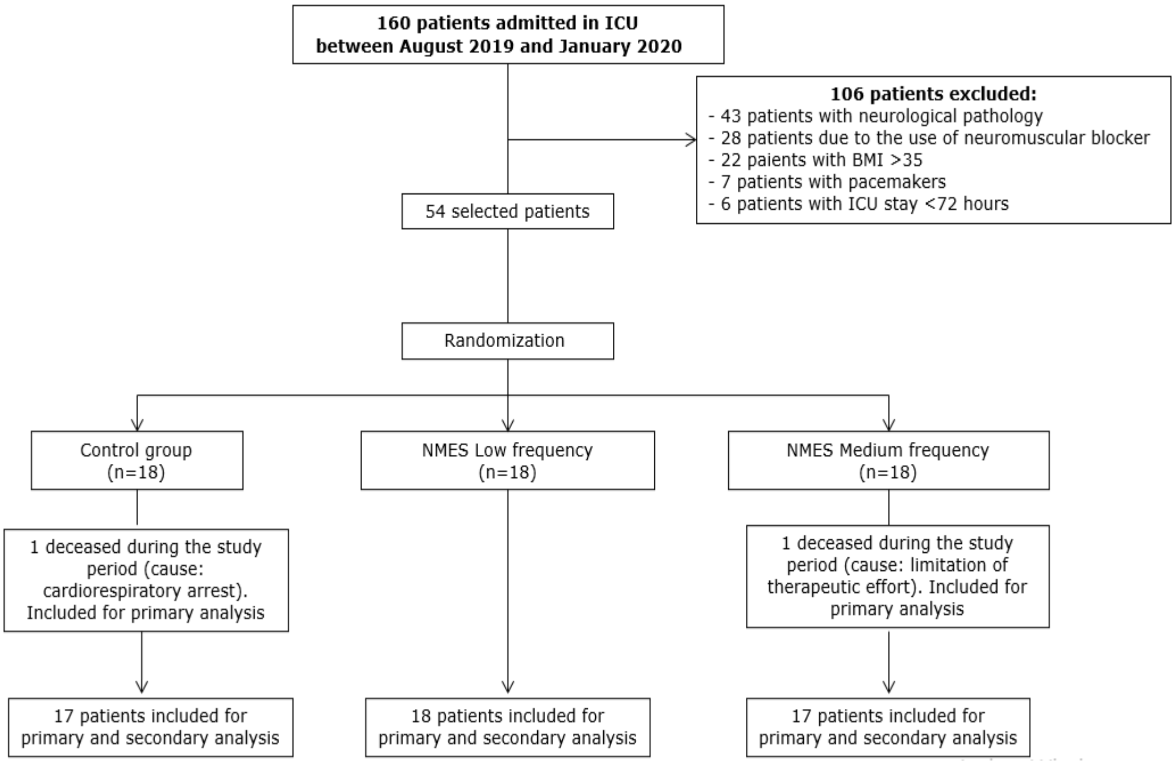

Supplemental Figure S1. CONSORT Flowchart displaying participant recruitment, randomization and data analysis. BMI: body mass index; NMES: neuromuscular electrical stimulation; ICU: Intensive Care Unit.

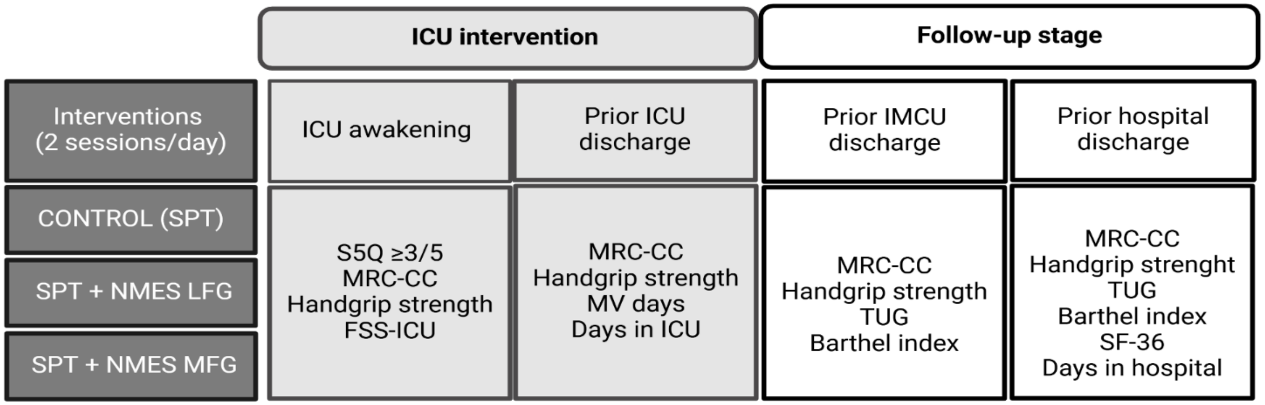

Supplemental Figure S2. Experimental design: ICU intervention and follow-up stages. FSS-ICU: Functional Status Score for the Intensive Care Unit; ICU: Intensive Care Unit; IMCU: Intermediate Care Unit; SPT: standard physical therapy; CONTROL: only SPT (group); LFG: low-frequency (group); MFG: medium-frequency (group); MRC-SS: Medical Research Council-Sum Score; MV: mechanical ventilation; NMES: neuromuscular electrical stimulation; S5Q: Standardized Five Questions; SF-36: Short Form 36; TUG: Timed Up and Go Test.
